# Lineage and Sublineage Analysis of Human Papillomavirus Types 66 and 68 in Iran During 2021–2023: A Cross‐Sectional Study

**DOI:** 10.1002/hsr2.70921

**Published:** 2025-07-09

**Authors:** Sima Taherkhani, Mohammad Shabanpour, Zabihollah Shoja, Somayeh Jalilvand

**Affiliations:** ^1^ Department of Virology, School of Public Health Tehran University of Medical Sciences Tehran Iran; ^2^ Department of Virology Pasteur Institute of Iran Tehran Iran

**Keywords:** HPV 66, HPV 68, human papillomavirus, lineage, and sublineage

## Abstract

**Background and Aims:**

Knowing the distribution of HPV lineages and sublineages can provide more information on this virus's epidemiology, evolution, and pathogenicity. The present study intends to investigate the sequence variations of the E6 gene to know the distribution of lineages and sublineages of HPV 66 and 68 types in Iran.

**Methods:**

Seventy‐one HPV 66 and 59 HPV 68‐positive samples were investigated to identify their lineage/sublineages by hemi‐nested PCR and sequencing.

**Results:**

Most of the HPV 66‐infected samples belonged to lineage B (77.5%) and the remaining (22.5%) were classified as lineage A. For sublineage classification, 22.5%, 12.7%, and 64.8% were sublineages A1, B1, and B2, respectively. Most of the HPV 68‐positive samples were classified as lineage C (66.1%), followed by lineage A (25.4%), lineage D/E (6.8%), and lineage F (1.7%). According to detected sublineages, C1 was dominant (62.7%), followed by A1 (22%), D/E (6.8%), A2 (3.4%), C2 (3.4%), and F2 (1.7%). No statistically significant differences were observed concerning HPV 66 or 68 distinct lineages by histology/cytology status.

**Conclusion:**

Our results showed that the B2 sublineage of HPV 66 and the C1 sublineage of HPV 68 were dominant in Iranian women. However, to elucidate the role of HPV 66 and 68 lineages in the pathogenicity risk of these two types, more studies with larger sample sizes are required.

## 1 Introduction

Human papillomavirus (HPV) is a group of nonenveloped epitheliotropic DNA viruses considered the most common sexually transmitted viral infection globally [1]. Although more than 440 different HPV types have been identified, almost 40 HPV types can infect the anogenital areas [2]. Human infection with high‐risk HPV types (16, 18, 31, 33, 35, 39, 45, 51, 52, 56, 58, 59, 66, and 68) causes cervical cancer [3, 4, 5]. Interestingly, infection with high‐risk HPV types is common in women, and most of these infections are cleared by the immune system within 6–24 months after the infection is established. In a few cases, these infections can persist and progress to high‐grade dysplasia, which may eventually lead to invasive cervical cancer [2].

Cervical cancer is the fourth leading cause of cancer in women worldwide. In 2022, 662,301 women were diagnosed with uterine cervical cancer, and 348,874 women have died from cervical cancer worldwide [6].

Papillomaviruses (PVs) are classified based on the DNA sequence of the L1 gene [7]. These viruses are divided into genera, species, and types based on 60%, 60%–70%, and 71%–89% identity in the L1 gene, respectively [8]. Within a given type, lineage and sublineage are considered for those HPVs that differ by 1%–10% and 0.5%–1% through the genome, respectively. HPV 66 is classified into A1, A2, and B (sub)lineages. HPV 68 is designated to seven distinctive lineages including A, B, C, D, E, F, and G. Each of HPV 68 lineages A, C, D, and F are divided into two different sublineages including A1, A2, C1, C2, D1, D2, F1, and F2 [8].

While the spreading of HPV types is well‐identified in uterine cervical samples among Iranian women [9], there is much less known regard for the HPV lineages and sublineages distributed in this area. Knowing the distribution of HPV lineages and sublineages can provide more information on this virus's epidemiology, evolution, and pathogenicity.

Until now, no studies have been found in Iran that characterized the lineages and sublineages of HPV 66 and 68. In this regard, the present study intends to investigate the sequence variations of the E6 gene to know the distribution of lineages and sublineages of these two types in Iran.

## 2 Materials and Methods

### 2.1 Study Population

A cross‐sectional study was planned from 2021 to 2023 to characterize the sequence variations of HPV 66 and 68 E6 genes. To find HPV 66 and 68‐positive samples, 1000 liquid‐based cervical (LBC) specimens (BD SurePath, Ireland) which were reported as HPV positive collected from several laboratories in Tehran were screened for these two types. Four‐hundred and sixty‐five formalin‐fixed paraffin‐embedded (FFPE) samples from different histological stages of premalignant and malignant cervical samples were also obtained from the archives of three hospitals in Tehran and previously screened for HPV and HPV typing. Finally, 71 HPV 66 and 59 HPV 68‐positive samples were investigated to identify their lineage/sublineages in this study.

This study was approved by the local ethical committee of the Tehran University of Medical Sciences (IR.TUMS.SPH.REC.1402.138).

### 2.2 Lineage/Sublineage Analysis of HPV 66 and 68 Based on the E6 Gene

The genomic DNA from FFPE samples was extracted by phenol‐chloroform assay, according to the previously published procedure [10]. The DNA isolation from liquid‐based cervical samples was carried out by the High Pure Viral Nucleic Acid Kit (Roche Diagnostics GmbH, Roche Applied Science) according to the manufacturer's instructions. The complete E6 gene of HPV 66 (nt 102‐569) and HPV 68 (nt 102–569) was amplified by hemi‐nested PCR with the following type‐specific primer pairs: 66‐E6F (AAAGGCAGCCTGTTGTGCC), 66‐E6R1 (GCTGTCCAATTGCTCATTGCAT), 66‐E6R2 (ATTGTAGGTCAATTTCCGTTTG), 68‐E6F (ATGGCGCTATTTCACAACCC), 68‐E6R1 (GGTGATTAACTGCATGGTCGG), and 68‐E6R2 (CTGCATGGTCGGGTTCATCTA) to obtain 572 and 620 amplicon sizes, respectively.

The PCR reaction was achieved in a 50 μL reaction mixture, including a 2× PCR master mix (AMPLIQON, Ampliqon A/s, Denmark), 10 pmol of each primer, and a DNA template. The PCR thermal cycling conditions were as follows: an initial denaturation for 5 min at 95°C, 35 cycles of 95°C for 30 s, 55°C for 50 s, and 72°C for 50 s (first round) and 35 cycles of 95°C for 25 s, 55°C for 40 s, and 72°C for 40 s (second round), respectively. A reaction mixture lacking DNA template, as a negative control, and a reaction mixture of positive control for HPV 66 or HPV 68 was run in every set of PCR runs. Positive controls were samples that previously were genotyped and confirmed to be infected with HPV 66 or 68.

To know HPV 66 and 68 (sub)lineages, the PCR products were sequenced using BigDye Terminator v3.1 Cycle Sequencing Kit and a 3130 Genetic Analyzer Automated Sequencer as specified by Applied Biosystems manuals. To classify lineages and sublineages of the studied samples, sequences were aligned to reference sequences with accession numbers U31794, EF177188, and EF177187 for HPV66 and DQ080079, KC470269, KC470270, FR751039, KC470274, KC470275, KC470276, KC470277, KC470279, and KC470281 for HPV 68. The phylogenetic tree was constructed by the maximum likelihood method and the Kimura 2‐parameter model using the Mega software version 11. The reliability was evaluated by the calculation of bootstrap with 1000 replicates.

### 2.3 Statistical Analysis

The statistical analysis was executed by the Mantel‐Haenszel test (Epi Info 7, Statistical Analysis System Software), and the *p* value (two‐sided) was considered statistically significant when it was less than 0.05.

## 3 Results

In total, 71 HPV 66‐infected cervical specimens (67 LBC and 4 FFPE samples) were included in this study. For HPV 68, 59 samples, including 57 LBC and 2 FFPE specimens were analyzed.

**Figure 1:**
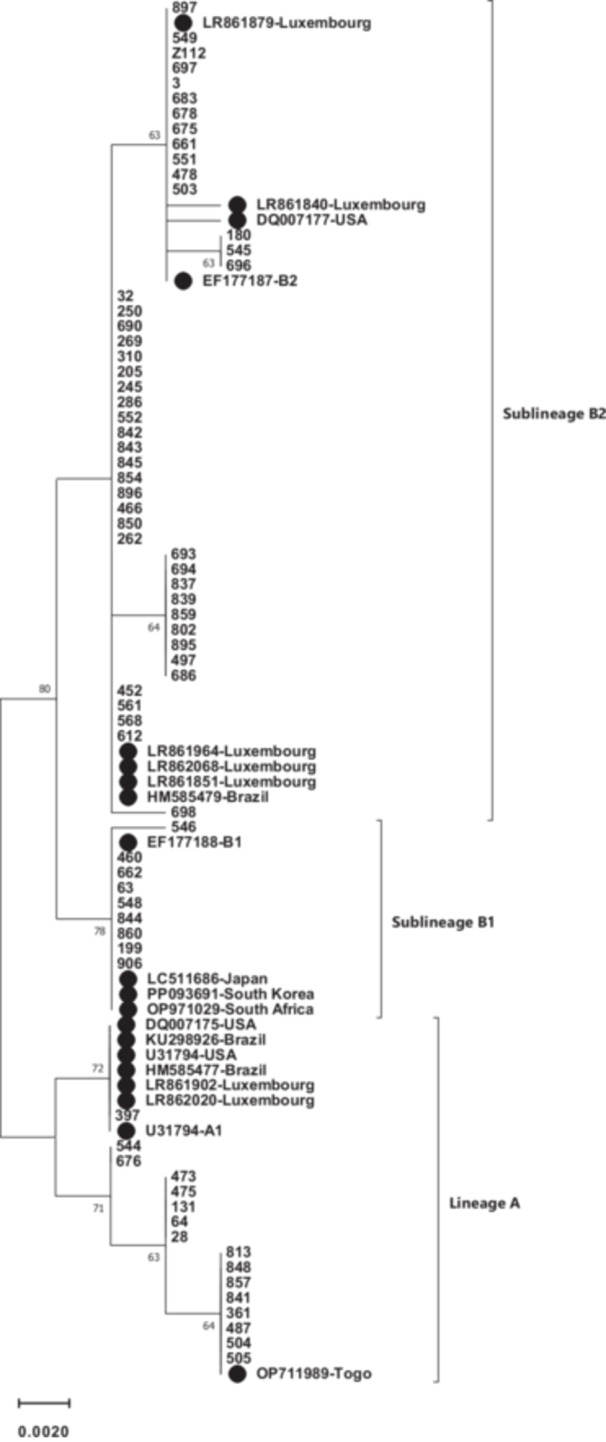
Phylogenetic analysis of the HPV 66 E6 gene, which was constructed in MEGA11 by the Maximum Likelihood method based on the Kimura 2‐parameter model. The reliability of the tree was tested using the bootstrap method with 1000 replicates, and the threshold of > 50 was considered to show in the tree. The reference sequences were shown by a black circle.

The whole E6 sequence (nt 102–569) of 71 HPV 66‐infected samples was compared to the HPV 66 reference sequence (GeneBank accession number U31794). Phylogenetic tree analysis was shown that the most of studied samples belonged to lineage B (77.5%) and the remaining them (22.5%) were classified as lineage A (Figure 1). Regard to sublineage classification, 22.5%, 12.7%, and 64.8% were A1, B1, and B2 sublineages, respectively.

As shown in Table 1, the nucleotide substitutions were detected in 70 samples. At 12 nucleic acid positions (T108C, T192G, T221G, C234T, C264T, C278T, A378G, C409T, G437A, A497C, G539C, and T551C) these changes were occurred. Among 12‐point mutations found in this study, eight changes were silent mutations (T221G, C234T, C264T, C278T, G437A, A497C, G539C, and T551C) and four substitutions were missense mutations (T108C, T192G, A378G, and C409T) which led to amino acid changes at positions of S3P, L31V, S93G, and S103L, respectively. The most frequent amino acid substitution was S93G, which was detected among 55 samples (77.5%). Other amino acid changes, including S3P, L31V, and S103L were found in 12.7%, 18.3%, and 1.4% of samples, respectively. Regarding different amino acid substitutions, no statistically significant differences were found between groups of normal and premalignant/malignant. However, the frequency of amino acid substitutions at positions of S93G and L31V were different in the two studied groups as S93G was more common in normal than premalignant/malignant groups (78.7% vs. 60%) and L31V was less frequent in normal samples in comparison to premalignant/malignant samples (14.7% vs. 40%). However, the differences did not reach statistically significant levels (*p* = 0.2 and 0.057, respectively).

**Table 1:** HPV 66 sublineages identified based on the E6 (102–569) gene in normal, premalignant, and invasive cervical cancer samples of Iranian women.

Sublineages (accession number)	Nucleotide substitution patterns	Patterns of amino acid changes	Acid nucleic substitutions												Studied groups		
			1	1	2	2	2	2	3	4	4	4	5	5	Normal/CI	CIN II–III/ICC	Total *N* = 71
			0	9	2	3	6	7	7	0	3	9	3	5	N I		
			8	2	1	4	4	8	8	9	7	7	9	1	*N* = 61	*N* = 10	
			T	T	T	C	C	C	A	C	G	A	G	T	*N* (%)	*N* (%)	*N* (%)
A (U31794)	—	—	—	—	—		—		—	—	—	—	—	—	1 (1.6)		1 (1.4)
A (this study)	C234T/C278T	—	—	—	—	T	—	T	—	—	—	—	—	—	2 (3.3)	—	2 (2.8)
A (this study)	T192G/C234T	L31V	—	G	—	T	—	—	—	—	—	—	—	—	2 (3.3)	3 (30.0)	5 (7.0)
A (this study)	T192G/C264T/A497C	L31V	—	G	—	—	T	—	—	—	—	C	—	—	7 (11.5)	—	8 (11.3)
B1 (EF177188)	T108C/C234T/C264T/A378G	S3P/S93G	C	—	—	—		—	G	—	—	—	—	—	7 (11.5)	1 (10.0)	8 (11.3)
B1 (this study)	T108C/C234T/C264T/A378G/C409T	S3P/S93G/S103L	C	—	—	T	T	—	G	T	—	—	—	—	1 (1.6)	—	1 (1.4)
B2 (EF177187)	C234T/C264T/A378G/G539C/T551C	S93G	—	—	—	T	T	—	G	—	—	—	C	C	10 (16.5)	2 (20.0)	12 (16.9)
B2 (this study)	C234T/C264T/A378G	S93G	—	—	—	T	T	—	G	—	—	—	—	—	5 (8.2)	—	5 (7.0)
B2 (this study)	T221G/C234T/C264T/A378G/T551C	S93G	—	—	G	T	T	—	G	—	—	—	—	C	8 (13.1)	1 (10.0)	9 (12.7)
B2 (this study)	C234T/C264T/A378G/T551C	S93G	—	—	—	T	T	—	G	—	—	—	—	C	14 (22.9)	2 (20.0)	16 (22.6)
B2 (this study)	C234T/C264T/A378G/G437A/G539C/T551C	S93G	—	—	—	T	T	—	G	—	A	—	C	C	3 (4.9)	—	3 (4.2)
B2 (this study)	C234T/C264T	—	—	—	—	T	T	—	—	—	—	—	—	—	1 (1.6)	—	1 (1.4)
			**Amino acid changes**														
			S	L					S	S							
			3	3	—	—	—	—	9	1	—	—	—	—			
			P	1					3	0							
				V					G	3							
										L							

*Note:* The accession numbers of reference sequences were shown in the parenthesis.

Abbreviations: CIN, cervical intraepithelial lesions; ICC, invasive cervical cancer.

The E6 sequence analysis of 71 HPV 66‐positive samples indicated that 12 and 5 different nucleotide and amino acid substitution patterns were observed in this study. Twelve acid nucleic change patterns were included no changes; C234T/C278T; T192G/C234T; T192G/C264T/A497C; T108C/C234T/C264T/A378G; T108C/C234T/C264T/A378G/C409T; C234T/C264T/A378G/G539C/T551C; C234T/C264T/A378G; T221G/C234T/C264T/A378G/T551C; C234T/C264T/A378G/T551C; C234T/C264T/A378G/G437A/G539C/T551C; and C234T/C264T. Five different amino acid substitution patterns were as follows: no amino acid changes; L31V; S3P/S93G; S3P/S93G/S103L; and S93G that were found in 4 (5.6%), 13 (18.3%), 8 (11.3%), 1 (1.4%), and 45 (63.4%) samples, respectively.

As indicated in Table 2, no statistically significant differences were observed between histology/cytology status and HPV 66 lineages (*p* = 0.15).

**Table 2:** The frequency of HPV 66 lineages stratified by histology/cytology status in cervical samples of Iranian women.

Histology/cytology status		Lineage A *N* (%)	Lineage B *N* (%)	Total *N* (%)	*p* value
	Normal/CIN I	12 (19.7)	49 (80.3)	61 (100)	
	CIN II–III/ICC	4 (40.0)	6 (60.0)	10 (100)	0.157
	**Total**	16 (22.5)	55 (77.5)	71 (100)	

Abbreviations: CIN, cervical intraepithelial lesions; ICC, invasive cervical cancer.

**Figure 2:**
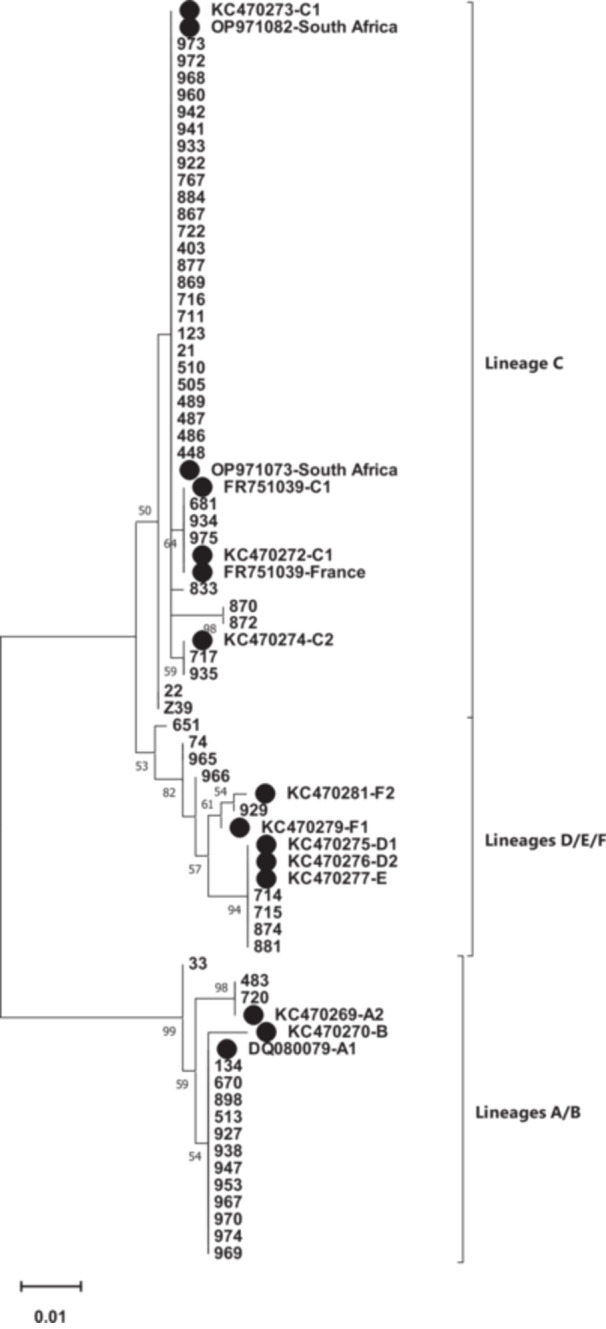
Phylogenetic analysis of the HPV 68 E6 gene, which was constructed in MEGA11 by the Maximum Likelihood method based on the Kimura 2‐parameter model. The reliability of the tree was tested using the bootstrap method with 1000 replicates, and the threshold of > 50 was considered to show in the tree. The reference sequences were shown by a black circle.

The full E6 sequence (nt 102–569) of 59 HPV 68‐positive samples was aligned against the HPV 68 reference sequence (GeneBank accession number DQ080079). As shown in Figure 2, most of the samples were classified to lineage C (66.1%), followed by lineage A (25.4%), lineages D/E (6.8%), and lineage F (1.7%). According to detected sublineages, C1 was dominant (62.7%), followed by A1 (22%), D/E (6.8%), A2 (3.4%), C2 (3.4%), and F2 (1.7%).

In total, 37 point mutations happened at the complete E6 gene of HPV 68 (Table 3). These mutations were observed in 47 samples (79.7%), and the remaining samples were without any mutations. Among 37 nucleotide substitutions, mutations at locations of A186G, C255T, T258A/G259A, T287A, A399G, T406C/G407A, A420T, T438A, C466A, A467T/G, A482T, and T556C were result in amino acid changes at positions of T29A, S50G, C53N/Y, F62L, N100D, L102S, S107C, C113S, T122N, T123S/A, L127F, and I152T, respectively. The amino acid substitution of C53N/Y was the most common mutation in the studied samples, that was found among 47 samples (79.7%). Each of the changes S50G, F62L, S107C, T122N, T123S/A, L127F, and I152T were detected in 44 samples (74.6%). Other mutations including T29A, N100D, L102S, and C113S were occurred among 3.4%, 69.5%, 72.9%, and 71.2% of samples, respectively.

**Table 3:** HPV 68 sublineages identified based on the E6 (102–569) gene in cervical samples of Iranian women.

Sublineages	Acid nucleic substitutions																																						Studied groups		
	1	1	1	2	2	2	2	2	2	2	2	2	3	3	3	3	3	3	3	3	4	4	4	4	4	4	4	4	4	4	4	5	5	5	5	5	5	5	Normal/CIN I	CIN II–III/ICC	Total
	7	8	9	0	4	5	5	5	5	6	7	8	0	0	2	2	4	6	6	9	0	0	2	2	3	4	4	6	6	6	8	1	3	5	6	6	6	7			
	0	6	4	3	9	1	5	8	9	6	2	7	2	8	0	7	7	2	5	9	6	7	0	6	8	3	6	6	7	8	2	8	6	6	0	3	6	5	*N* = 53	*N* = 6	*N* = 59
(Accession number)	T	A	C	T	A	T	C	T	G	G	A	T	A	T	T	C	G	A	T	A	T	G	A	C	T	A	A	C	A	A	A	G	G	T	T	A	A	T			
A1 (DQ080079)	—	—	—	—	—‐	—	—	—	—	—	—	—	—	—	—	—	—	—	—	—	—	—	—	—	—	—	—	—	—	—	—	—	—	—	—	—	—	—	11 (20.6)	1 (16.7)	12 (20.2)
A2 (KC470269)	—	G	—	C	—	—	—	—	A	—	—	—	—	A	—	—	—	—	—	—	—	—	—	—	—	—	—	—	—	—	—	—	—	—	—	—	—	‐‐‐	2 (3.8)	—	2 (3.4)
A2 (this study)	—	—	—	—	—	—	—	—	A	—	—	—	—	—	—	—	—	—	—	—	—	—	—	—	—	—	—	—	—	—	—	—	—	—	—	—	—	—	—	1 (16.7)	1 (1.7)
B (KC470270)	—	—	G	—	—	—	—	—	—	—	—	—	—	—	—	—	—	—	—	—	—	—	—	T	—	—	—	—	G	—	—	—	—	—	—	—	—	—	—	—	—
C1 (FR751039)	C	—	—	—	G	—	T	A	A	A	G	A	—	—	G	—	A	—	A	G	C	A	T	—	A	T	T	A	T	T	T	C	A	C	G	G	‐‐‐	A	1 (1.9)	2 (33.2)	3 (5.1)
C1 (this study)	C	—	—	—	G		T	A	A	A	G	A	—	—	—	—	A	—	A	G	C	A	T	—	A	T	T	A	T	T	T	C	A	C	G	G	‐‐‐	A	24 (45.3)	1 (16.7)	25 (42.4)
C1 (this study)	C	—	—	—	G	—	T	A	A	A	G	A	—	—	—	—	A	—	A	G	C	A	T	—	A	T	T	A	T	T	T	C	‐‐‐	C	G	G	‐‐‐	A	2 (3.8)	—	2 (3.4)
C1 (this study)	C	—	—	—	G	—	T	A	A	A	G	A	—	—	—	—	—	—	—	G	C	A	T	—	—	—	T	A	T	T	T	C	A	C	G	G	—	A	2 (3.8)	—	2 (3.4)
C1 (this study)	C	—	—	—	G	—	T	A	A	A	G	—	—	—	—	—	A	—	A	G	C	A	T	—	A	T	T	A	T	T	T	C	A	C	G	G	—	A	1 (1.9)	—	1 (1.7)
C1 (this study)	—	—	—	—	G	—	T	A	A	A	G	A	—	—	—	—	A	—	A	G	C	A	T	T	A	T	T	A	T	T	C	C	—	C	G	G	G	A	2 (3.8)	—	2 (3.4)
C1 (this study)	—	—	—	—	G	—	T	A	A	A	G	A	—	—	—	—	A	—	A	G	C	A	T	T	A	—	T	A	T	T	C	C	—	C	G	—	G	A	1 (1.9)	—	1 (1.7)
C1 (this study)	—	—	—	—	G	—	—	A	A	A	G	A	—	—	—	—	A	—	A	G	C	A	T	—	A	T	T	A	T	T	C	C	—	C	G	G	G	A	1 (1.9)	—	1 (1.7)
C2 (KC470274)	—	—	—	—	G	—	T	A	A	A	G	A	—	—	—	—	A	—	A	G	C	A	T	—	A	T	T	A	T	T	T	C	A	C	G	G	—	A	1 (1.9)	1 (16.7)	2 (3.4)
D1 (KC470275)	—	—	—	—	G	—	T	A	A	A	G	A	C	—	—	A	A	C	A	G	C	A	T	T	A	—	T	A	T	T	C	C	—	C	G	G	G	A	4 (7.5)	—	4 (6.8)
D2 (KC470276)	—	—	—	—	G	—	T	A	A	A	G	A	C	—	—	A	A	C	A	G	C	A	T	T	A	—	T	A	T	T	C	C	—	C	G	G	G	A			
E (KC470277)	—	—	—	—	G	—	T	A	A	A	G	A	C	—	—	A	A	C	A	G	C	A	T	T	A	—	T	A	T	T	C	C	—	C	G	G	G	A			
F1 (KC470279)	—	—	—	—	G	C	T	A	A	A	G	A	—	—	—	—	A	C	A	G	C	A	T	T	A	—	T	A	T	T	C	C	—	C	G	—	G	A	—	—	—
F2 (KC470281)	—	—	—	—	G	C	T	A	A	A	G	A	—	—	—	—	A	C	A	G	C	A	T	T	A	—	T	A	T	G	C	C	—	C	G	G	G	A	1 (1.9)	—	1 (1.7)
	**Amino acid changes**																																								
		T	D				S	C	C53Y			F								N	L	L	S		C			T	T	T	L			I							
	—	2	3	—	—	—	5	53N		—	—	6	—	—	—	—	—		—	1	1	1	1	—	1	—	—	1	1	1	1	—	—	1	—	—	—	—			
		9	1				0					2								0	02S	02S	0		1			2	2	2	2			5							
		A	E				G					L								0			7		3			2	3	3	7			2							
																				D			C		S			N	S/A	S/A	F			T							

*Note:* The accession numbers of reference sequences were shown in the parenthesis.

Abbreviations: CIN, cervical intraepithelial lesions; ICC, invasive cervical cancer.

The results of this study were shown that 14 different nucleotide substitution patterns (Table 3) and five amino acid change patterns were detected. These five amino acid substitution patterns were included no change (20.3%); T29A/C53Y (3.4%); S50G/C53N/F62L/N100D/L102S/S107C/C113S/T122N/T123S/L127F/I152T (69.5%); C53Y/F62L/S107C//T122N/T123/L127F/I152T (3.4%); and S50G/C53Y/F62L/S107C C113S/T122N/T123/L127F/I152T (1.7%).

**Table 4:** The frequency of HPV 68 lineages stratified by histology/cytology status in cervical samples of Iranian women.

Histology/cytology status		Lineage A *N* (%)	Lineage C *N* (%)	Lineage D/E/F *N* (%)	Total *N* (%)	*p* value
	Normal/CIN I	13 (24.5)	35 (66.0)	5 (9.5)	53 (100)	0.69
	CIN II‐III/ICC	2 (33.3)	4 (66.7)	0 (0.0)	6 (100)	
	**Total**	15 (25.4)	39 (66.1)	5 (8.5)	59 (100)	

Abbreviations: CIN, cervical intraepithelial lesions; ICC, invasive cervical cancer.

Stratification by histology/cytology status showed that no statistically significant differences were observed concerning HPV 68 distinct lineages (Table 4).

## 4 Discussion

It is well‐documented that the spread of HPV 16, 18, and 58 (sub)lineages are different in the world, which is related to the evolution of different host ethnicities [11, 12, 13, 14]. However, such a relationship is not well‐known for other high‐risk types of HPV. At this point, this study was conducted to characterize the lineages and sublineages of HPV 66 and 68 in the Iranian population.

Our findings indicated that 77.5% and 22.5% of HPV66‐positive samples belonged to lineages B and A, respectively. With regard to detected sublineages, B2 was the most common sublineages (64.8%) followed by A1 (22.5%) and B1 (12.7%) (Figure 1 and Table 1). This result is consistent with the findings of the study in Italy that reported lineage B and A among 60.9% and 30.1% of studied samples [15]. Several studies have shown that the distribution of lineages and sublineages of HPV 66 was different in the world. The result of a study from Costa Rica reported that lineage A and B were detected in 53% and 47% of samples, respectively [16]. In another study conducted in Costa Rica and Rwanda, it is shown that lineage A and B were found in 47.7% and 52.3% of Costa Rican women and 63.6% and 33.4% of Rwandan women, respectively [17]. In Luxembourg, lineages A and B were detected in 50% and 50% of samples [18]. The data from Chile indicated that 55.6% and 44.4% of specimens belonged to A and B lineages, respectively. However, lineage A and B were shown in 6.4% and 93.3% of samples from Shanghai [19]. Looking at the distribution of distinct sublineages of HPV66, it was shown that B2 was more prevalent than B1 among Shanghainese and Costa Rican women [17, 19] as the same as Iranian women.

Among 12 point nucleotide changes found in this study, eight changes were transition (C234T, C264T, C278T, G437A, T108C, A378G, C409T, and T551C) and four substitutions were transversion (T221G, A497C, G539C, and T192G) mutations. Four out of twelve nucleotide changes including T108C, T192G, A378G, and C409T result in amino acid substitutions at S3P, L31V, S93G, and S103L positions, respectively. Among amino acid changes, S93G was dominant and detected in 77.5% of samples, followed by L31V (18.3%), S3P (12.7%), and S103L (1.4%) of samples. However, no differences were observed between the normal and premalignant/malignant groups by amino acid substitutions (Table 2).

It has been suggested that amino acid substitutions in the E6 and E7 genes can confer higher oncogenesis potential compared to wildtype [20]. Indeed, the increased expression of several host genes such as genes involve in cell cycle, several signaling pathways, and migration are found by HPV 18 E6 of African variants [21]. However, functional analysis of HPV 66 E6 variants (wild type vs. S93G mutant) to ability for p53 degradation was shown that no differences were observed between these two variants [22].

Our results indicated that lineage B was more common in both studied groups and no statistically significant differences were observed in this regard (Table 3). This finding can be due to the low sample size that does not let us to find main differences.

The variant analysis on HPV68‐positive samples revealed that most of the samples were classified to lineage C (66.1%), followed by lineage A (25.4%), lineages D/E (6.8%), and lineage F (1.7%). With regard to identified sublineages, C1 was dominant (62.7%), followed by A1 (22%), D/E (6.8%), A2 (3.4%), C2 (3.4%), and F2 (1.7%) (Figure 2). A study in America had shown that the distribution of HPV 68 lineages was descending included E (47.2%), C (22.6%), F (18.1%), G (4.2%), A (3.8%), D (2.6%), and B (1.5%) [23]. Another study from Costa Rica identified lineages A and B in 29.9% and 70.1%, respectively [16].

From 37 mutations, 18 (48.6%) and 19 (51.4%) mutations were transition and transversion changes, respectively. Twelve mutations at locations of A186G, C255T, T258A/G259G, T287A, A399G, T406C/G407A, A420T, T438A, C466A, A467T/A468T(G), A482T, and T556C were led to amino acid changes at positions of T29A, S50G, C53N/Y, F62L, N100D, L102S, S107C, C113S, T122N, T123S/A, L127F, and I152T, respectively; among which the amino acid changes at positions of S50G, C53N, F62L, N100D, L102S, S107C, C113S, T122N, T123S/A, L127F, and I152T were frequently detected in our studied samples.

Previous studies concerning variant analysis of several high‐risk HPVs indicated that distinct (sub)lineages are frequent in Iranian women including lineage D of HPV 16, sublineage A4 of HPV 18, lineage A of HPV 31, sublineage A1 of HPV 39, lineage B of HPV 45, lineage A of HPV 51, lineage A of HPV 52, lineage B of HPV 56, sublineage B1 of HPV 58 and lineage B of HPV 59 [24, 25, 26, 27, 28, 29, 30]. The results of this study revealed that sublineage B2 of HPV 66 and sublineage C1 of HPV 68 are dominant in Iran.

One limitation of this study was the small sample size that did not allow us to find whether there is an association between HPV 66 or 68 lineages with histology status or not. Also, lineage analysis was done based on E6 gene which is a small proportion of the whole genome.

In conclusion, our results showed that the B2 sublineage of HPV 66 and the C1 sublineage of HPV 68 were dominant in Iranian women. It is highly recommended that in the future studies, the distribution of HPV 66/68 (sub)lineages would be investigated using the whole genome sequencing in different geographical parts of Iran. Also, to elucidate the role of HPV 66 and 68 lineages in the pathogenicity risk of these two types, more studies with larger sample sizes are required to analyze.

## Author Contributions

**Sima Taherkhani:** formal analysis, investigation, and project administration. **Mohammad Shabanpour:** formal analysis, investigation, methodology, and software. **Zabihollah Shoja:** data curation, formal analysis, methodology, validation, visualization, and writing – original draft. **Somayeh Jalilvand:** conceptualization, data curation, funding acquisition, resources, supervision, writing – review and editing.

## Ethics Statement

Our research was conducted ethically according to the World Medical Association Declaration of Helsinki. We declare that informed consent was obtained from all study subjects and the study was approved by the local ethical committee of Tehran University of Medical Sciences (IR.TUMS.SPH.REC.1402.336).

## Conflicts of Interest

The authors declare no conflicts of interest.

## Transparency Statement

The corresponding author, Somayeh Jalilvand, affirms that this manuscript is an honest, accurate, and transparent account of the study being reported; that no important aspects of the study have been omitted; and that any discrepancies from the study as planned (and, if relevant, registered) have been explained.

## Data Availability

Our sequences were deposited in Nucleotide—NCBI (http://nih.gov) with the following accession numbers: PQ417145‐PQ417274.
